# An opposed matched field IMRT technique for prostate cancer patients with bilateral prosthetic hips

**DOI:** 10.1120/jacmp.v13i1.3347

**Published:** 2012-01-05

**Authors:** Shahin Fattahi, Orest Z. Ostapiak

**Affiliations:** ^1^ Department of Medical Physics Juravinski Cancer Centre Hamilton ON Canada L8V 5C2

**Keywords:** radiation therapy, prostate cancer, bilateral hip prostheses, IMRT

## Abstract

Intensity‐modulated radiation therapy (IMRT) has gained wide‐spread use for treating patients with prostate cancer, yet developing a plan for patients with bi‐lateral metal hip prostheses implants may be challenging. The high atomic number of the metallic hips not only gives rise to streak artifacts that obscure anatomy but also attenuates laterally directed fields by a significant amount that cannot be reliably ascertained from the CT dataset. A common approach to planning directs five IMRT fields such that incidence through the metal hips is avoided. While this technique generally gives adequate PTV coverage, it may escalate the rectal dose if beams, which would otherwise be incident from a lateral direction, are angled toward a posterior direction in order to avoid the prosthesis. In this work, we propose and investigate a new technique which alleviates this problem by introducing asymmetric opposed fields that are edge‐matched along a plane that is tangent to the metal prostheses. With this approach, a posterior oblique field is oriented closer to the lateral direction but does not irradiate the ipsilateral prosthesis. The portion of the target eclipsed by the prosthesis is irradiated by the opposed matched anterior oblique field which, again, avoids the corresponding ipsilateral prosthesis. While the proposed technique may improve rectal sparing and PTV coverage, the dose along the match plane is sensitive to intrafraction motion. In the worse case of intrafraction motion perpendicular to the plane occurring in the time interval between the deliveries of successive fields of the opposed matched pair, the induced error is typically about 5 cGy per mm of target motion for a 200 cGy fraction. To reduce the induced error, several approaches to broadening the penumbra at the match plane were investigated and compared to conventional IMRT plans for three patients. Phantom measurements were performed to evaluate the effectiveness of these approaches. Match‐plane shifts of 4 mm in a single step, in two 2 mm steps, and in four 1 mm steps, were effective in reducing the worse case induced error to 2.8 cGy per mm. Imposing match‐plane shifts precludes the use of intensity modulation for the opposed matched field pairs. Therefore, we favor an approach whereby the opposed matched fields overlap by 4 mm. Since both fields contribute fluence to the overlap region, the worse case induced error was observed to be typically within 2.9 cGy per mm. In conclusion, the use of this technique should be considered for patients with bilateral metal hip implants who do not meet dose‐volume criteria by conventional IMRT techniques.

PACS number: 87.55.‐x

## I. INTRODUCTION

Intensity‐modulated radiation therapy (IMRT) has gained widespread acceptance as the technique of choice for prostate cancer patients undergoing radiotherapy. This is primarily due to the need for escalation of dose to the prostate which would otherwise be limited by toxicity to the rectum, bladder, and femoral heads with conventional radiotherapy. IMRT allows the design of dose distributions that are conformal to the target volume with dose gradients tailored to spare the organs at risk.

Across Canada, the age‐standardized rate for hip replacement for males in 2006–2007 was 75.6 per 100,000. Thus, it is not uncommon to encounter prostate cancer patients with bilateral metal hip prostheses (MHP).^(^ ^1^ ^)^

Planning radiotherapy treatments for these patients is complicated for three reasons associated with the high atomic number of the MHP. First, there are severe streak artifacts that obscure the prostate and critical organs at risk, making the delineation of these structures difficult. Second, CT numbers do not correspond to electron density near streak artifacts or within the MHP itself^(^ ^2^ ^)^ thereby potentiating dose calculation errors in these regions. Third, even if dose could be correctly calculated, a lateral field passing through the proximal MHP would be attenuated by between 10% and 64%^(^ ^3^ ^–^ ^9^ ^)^ and would therefore require a high degree of modulation and targeting precision.

The first complication is effectively dealt with by fusing an MRI scan to the planning CT in order to visualize the prostate. To overcome the latter two problems, it is common practice to arrange the beams so as to avoid the MHP in the beam's eye view (BEV).^(^ ^3^ ^,^ ^10^ ^)^ For patients with a unilateral MHP, this method can be implemented easily by aligning the lateral beam in either a posterior or anterior direction so as to avoid irradiation of the ipsilateral MHP without having to reduce the number of beams.^(^ ^10^ ^)^

**Figure 1:**
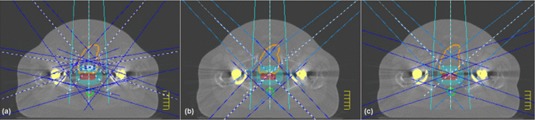
IMRT beam arrangements used for prostate irradiation: (a) 7‐field technique; (b) conventional 5‐field IMRT (CFIMRT) technique; (c) the opposed matched field (OMF) technique. The arrangements illustrated in (b) and (c) are used for patients with bilateral MHP. The two posterior oblique beams shown in (b) are also used in (c) but have been removed for clarity. The bladder (orange contour), rectum (green contour), CTV (red colorwash), PTV margin (blue colorwash), and metal hip prostheses (yellow colorwash) are indicated for reference. Note that the CT scans are obtained with a GE LightSpeed

For prostate patients with bilateral MHP (henceforth referred to simply as patients), radiotherapy planning is further complicated by the necessity to re‐align or eliminate both lateral beams. In the absence of MHP, prostate irradiation is commonly achieved using between 5 and 7 coplanar IMRT fields. A 7‐field IMRT technique is shown in Fig. 1(a). In cases with bilateral MHP, a 5‐field technique is commonly used whereby the beams are angled so as to avoid each MHP in the BEV of each field as shown in Fig. 1 (b). This method has been widely adopted for conformal radiotherapy of the prostate for these cases. The application of IMRT to this problem has been investigated by several authors. The earliest investigation used the common 5‐field technique in conjunction with IMRT^(^ ^3^ ^)^ (CFIMRT). It was found that the use of IMRT resulted in better rectal sparing than is possible with conventional fields. However, this approach redirects the lateral beams to aim from a more posterior direction in order to avoid the prosthesis, resulting in a greater volume of rectum irradiated. Therefore, the dose to the rectum is increased compared to the standard 7‐field IMRT technique. An alternative approach has been suggested^(^ ^11^ ^,^ ^12^ ^)^ whereby the beams incident through the MHP are modulated so as to uniformly irradiate the PTV. This approach relies on electronic portal imaging to determine the modulated fluence pattern required. By this approach, the compensated beams irradiate the PTV uniformly to within ±5%, but deliver a greater dose to in the regions adjacent to the prosthetic hips and are susceptible to substantial dose error from intrafraction motion. Recently, the use of a noncoplanar IMRT technique for one patient has been reported.^(^ ^13^ ^)^ This technique avoids incidence through the MHP by angling the couch and allowing select beams to cover only a portion of the PTV. This technique was able to spare the rectum over a clinically relevant dose range better than a coplanar 7‐field IMRT technique; however, the noncoplanar beams penetrate a greater depth of tissue and contribute greater dose to bladder and normal tissue than their coplanar counterparts.

In this paper, we introduce an opposed matched field (OMF) technique similar to one that has already been used for the treatment of head and neck cancer with the aim to spare the parotids.^(^ ^14^ ^)^

Despite these advantages, the introduction of a match plane between OMF pairs raises the concern for increased susceptibility of dose delivery errors due to intrafraction motion. In this work, the magnitude of this error is assessed using both measurements and calculations in a pelvic phantom. The effectiveness of adding a shifting junction (SJ) or an IMRT field overlap (O) to the OMF technique is investigated.

## II. MATERIALS AND METHODS

The objective of this investigation is to determine the suitability of the OMF technique for clinical use. As such, this work is organized into two parts. In the first part, we compare Pinnacle IMRT treatment plans using the four techniques (CFIMRT, OMF, OOMF, and SJOMF) for three patients. Evaluation of these techniques is based on DVH data corresponding to the rectum, bladder, CTV, and PTV. In the second part, the accuracy of the delivery system and the effect of intrafraction motion on dose in the vicinity of the isocenter are investigated by measuring the dose from an opposed matched pair along a line perpendicular to the match plane in a phantom.

Irrespective of technique, patients were scanned using a GE LightSpeed RT scanner (General Electric, Fairfield, CT) capable of extended Houndsfeld Units (HU) from −31,743 to +31,743 HU. The extended HU capability is somewhat effective in reducing the streak artifact due to the high density of the MHP compared to scanners restricted to reconstruction in the conventional range of −1,024 to +3,071 HU. A value of well over +3,071 HU is expected for metals such as titanium (roughly 8,000 HU) and stainless steel (roughly 12,000 HU)^(^ ^15^ ^)^ — materials commonly found in a MHP. All prostate contours at our center are delineated by a radiation oncologist on the CT dataset. On slices where organs are obscured by artifact, contours are interpolated from other slices. While an MRI scan would be preferable for visualizing the prostate under these circumstances, it is not readily available at our institution for this purpose.

To overcome the influence of streak artifacts on dose calculation, all soft‐tissue density (excluding MHP and bone) is overridden with unit density. We expect this density override to introduce a dosimetric error of less than 2% for patients of normal weight; however, care must be taken with patients that are either slim and muscular or obese since the override may result in an underdose or overdose for these respective body types by an amount greater than 3%. The MHP is also contoured for the purpose of visualization in a BEV, but its density is not overridden for two reasons. First, it is difficult to determine the effective density of a MHP from a CT scan due to the X‐ray hardening artifact. A MHP may be made from a combination of materials including cobalt chromium, ceramic zirconium, ceramic aluminum, Oxinium (Smith & Nephew, Mississauga, ON Canada), and stainless steel. Therefore, no a priori assumption may be made regarding the MHP composition and, hence, its density. Second, with our technique, no beam ray path intersects a MHP before irradiating the target. Therefore, density within the MHP is not important for dose calculation since the exit dose beyond a distal MHP is not a clinically important quantity.

All plans were developed for irradiation with the 6 MV beam of a Varian 21EX LINAC with 120 leaf Millennium MLC using the ${\text{Pinnacle}}^{3}$ version 8.0d radiotherapy treatment planning system (Philips Medical Systems, Cleveland, OH). Implementation of nonconventional techniques on alternative planning systems is contingent on the system allowing the planner to fix the jaw positions prior to optimization.

### A. IMRT technique description

#### A.1 Conventional 5‐field IMRT (CFIMRT) technique

The CFIMRT technique is commonly used by many institutions for treating patients with bilateral MHP.^(^ ^3^ ^)^ As implemented at our institution, the technique consists of anterior, right‐posterior‐oblique (RPO), left‐posterior‐oblique (LPO) coplanar fields, as well as right‐anterior‐oblique (RAO) and left‐anterior‐oblique (LAO) noncoplanar fields that are slightly angled for incidence from an inferior direction. All fields are oriented such that they all avoid the proximal MHP in their BEV. The collimators are rotated to block fully the proximal MHP while exposing the PTV. It should be noted that the gantry and collimator angles may vary depending on each patient's geometry and thickness, and the position of MHPs relative to the PTV. Also note that inverse planning was used.

#### A.2 Opposed matched field (OMF) technique

This technique can be viewed as a 5‐field technique with two additional opposed matched field pairs. To implement this technique, the 5‐field technique is designed as described above. Subsequently, two opposed matched field pairs are added. One pair consists of a LAO and RPO OMF pair, and the other consists of a RAO and LPO OMF pair. In a given pair, the gantry is adjusted such that the central axis of the field is tangent to the edge of the MHP eclipsing the target. For each field in the pair, the collimators are set so that one aligns with the central axis, while the other exposes the portion of the target not eclipsed by the proximal MHP. The OMF geometry is illustrated in Fig. 1(c). The advantages gained with this geometry over the 5‐field IMRT technique are two‐fold. First, one may expect a greater degree of rectal sparing since the prostate is irradiated from a more lateral direction than that achievable if each field is to irradiate fully the target volume. Second, one may expect improved dose uniformity within the PTV since the use of a match plane ensures that there are no regions of beam overlap or underlap.

#### A.3 Junction management techniques

It is well known that the introduction of a match plane in a radiotherapy treatment increases the possibility of over‐ or underdosing regions adjacent to the match plane. These errors may be due to collimator misalignments or intrafraction motion of the target volume.^(^ ^16^ ^–^ ^18^ ^)^ In particular, if the dose from one of the OMF falls off sharply at the match plane, then tissue translation across this plane that occurs in the time interval between the deliveries of the two OMF may result in a significant delivery error. In order to minimize this effect, one must both minimize the time interval and design dose profiles that fall off gradually in this region. The latter approach is also referred to as penumbra spoiling or junction feathering.^(^ ^16^ ^)^

#### A.4 Shifting junction opposed matched field (SJOMF) technique

In segmented field delivery, junction feathering can be achieved by shifting the match plane one or more times during the beam delivery. This creates a step‐wise fall off of the dose profiles across the match plane.^(^ ^16^ ^)^

To implement this technique, we first create a region of overlap across the match plane by opening the matched jaws on each opposed matched pair field by 2 mm. This results in a 4 mm wide overlap region at the isocenter that extends along the match plane. This width was chosen since it is roughly equal to the 80%–20% penumbra of a 6 MV field and could therefore be expected to influence the dose gradient, but is not so large as to overly restrict the positioning of the laterally directed opposed matched fields. Within the overlap region, the match plane is shifted by 4 mm using two MLC segments. It should be noted that in order to achieve a uniform dose profile across the match plane, the MLC leaves of each opposed matched field segment must overlap the nominal match plane by 0.7 mm at the isocenter.

It is important to note that OMF that are segmented in this way can no longer be included in the inverse planning optimization. Therefore, the only parameter that can be optimized with respect to these beams is weight. In the end, we found that the manual design of these shifting match plane segments was relatively time‐consuming.

#### A.5 Overlapping opposed matched field (OOMF) technique

An alternative approach to reducing dosimetric errors arising from the use of a match plane is to allow the OMF to overlap as outlined in the previous section but, rather than segment the fields manually, the fields are included in the IMRT optimization. The Pinnacle treatment planning system allows the user to fix the jaws' positions so that they are not altered over the course of the optimization. Our expectation is that each beam of the opposed matched pair contributes roughly equally to the dose in the overlap region. If this is the case, then sensitivity of dose error due to motion across the match plane would be correspondingly reduced by a factor of two. This approach offers advantages over the SJOMF technique. Namely, it is less labor‐intensive to implement, and it allows the OMF to be modulated by the inverse planning engine.

### B. Planning study

Three patients were selected from our institution's patient database. These patients were planned using the CFIMRT, OMF, SJOMF, and OOMF techniques. The optimization objectives used for each patient are set out in Table 1. The ROI optimization objectives were maintained for all patients and techniques, but the dose levels and weights were adjusted for each patient in order to minimize dose to the rectum and bladder. Note that for a given patient, a single set of optimization objectives was used for all the techniques. After optimization, the total number of MUs was adjusted by a small amount so that 98% of the PTV was covered by 95% of the prescription dose. This approach ensured a fair comparison among techniques.

**Table 1:** IMRT dose objectives.

*ROI*	*Volume (cc)*	*Objective*	*Target (cGy)*	*Weight (%)*	*a*
Bladder	190/527/115	Max EUD	4500/2490/4510	20/20/20	2/2/2
CTV	95/31/65	Max Dose	8000/8100/8100	80/50/50	
CTV		Min Dose	7700/7700/7680	100/100/100	
ORingPTV		Max Dose	7220/7220/7230	10/10/10	
ORingPTV		Max EUD	5700/5430/5640	10/10/10	3/3/3
Rectum	57/82/30	Max EUD	5350/3340/4560	20/20/20	2/1/1
RingPTV	167/168/133	Max Dose	7800/7800/7800	20/20/10	
RingPTV		Max Dose	7300/7400/7400	100/100/100	

Dose objectives used for IMRT optimization are set out corresponding to patients 1/2/3 respectively. The second column is not directly related to the assigned objectives but provides some indication of how anatomy differs among the patients. RingPTV refers to the volume contained within the PTV that excludes the CTV, and ORingPTV refers to the volume contained within a 1.5 cm margin encircling the PTV, but excluding the volume within the PTV. All other region of interest (ROI) labels are self‐explanatory. The equivalent uniform dose (EUD) objective is associated with the parameter a, whose value is tabulated in the last column.

### C. Dosimetric studies

**Figure 2:**
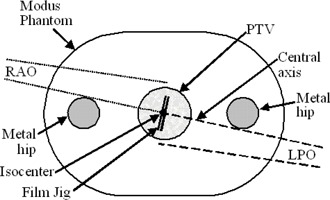
Schemetic illustration of an axial slice through the Modus phantom used in the dosimetric studies. The phantom measures 30 cm wide, 20 cm high, and 12 cm long. The schematic shows the geometric arrangement of opposed matched fields and film jig used for measuring field profiles.

In our dosimetric study, we used the cylindrical body phantom (Quasar, Modus Medical, London, ON, Canada) as shown in Fig. 2. Two prosthetic hips were set in gelatin and replaced the two lateral inserts of the phantom. The phantom with the hips inserted was CT‐scanned using a GE LightSpeed RT CT scanner. The DICOM images were then imported into Pinnacle. Extended CT numbers were used in Pinnacle and a density of $7\;g/{\text{cm}}^{3}$ (which is somewhat less than that of stainless steel, 7.48 g/cc, but more than that of titanium, 4.5 g/cc) was assigned to both contoured hip volumes. Anatomical regions representing the prostate, bladder, and rectum were also contoured. The OMF plan was then generated for the phantom and delivered on a Varian Clinac 21EX linac. This technique was not tested on linacs from other manufacturers; however, it is reasonable to expect that any system capable of step‐and‐shoot delivery could be used.

#### C.1 Dosimetric verification

In this section, the dose calculated by the TPS is verified against measurements in the phantom using film. The opposed matched pair consisting of the RPO field opposed matched to the LAO field were selected and delivered to this phantom. For the measurement of dose from each beam, calibrated EBT GAFCHROMIC film was used. The film was placed inside a custom‐made jig which was inserted into the central cavity of the phantom, as shown in Fig. 2. The jig was then rotated such that the plane of the film was perpendicular to central axis of the opposed‐matched pair. This measurement geometry is shown in Figure 2. The films were then scanned, and the profile from each beam was generated along a line passing through the isocenter and perpendicular to the match plane. Theses profiles were compared against the profiles computed by the TPS.

#### C.2 Sensitivity of dose at the match plane to intrafraction motion

To investigate the magnitude of dosimetric errors caused by intrafraction target motion, we irradiated the body phantom with the LAO field and applied shifts of various magnitudes perpendicular to the match plane prior to irradiating with the opposed matched RPO field. In Pinnacle, six points were generated in the transverse plane containing the isocenter and along the line perpendicular to the match plane of the LAO and RPO field. These points were separated by 1 mm and distributed on either side of the isocenter. By resetting the isocenter of one beam to one of these points, the effect of a target shift is simulated within Pinnacle.

To realize these same shifts on a Varian Clinac 21EX linear accelerator, the phantom was mounted on precision translation stages supported on the treatment couch, which allowed both vertical and lateral submillimeter adjustment of position. A calibrated 0.6 cc Farmer‐type ion chamber was placed into the phantom's central jig insert and aligned with the machine isocenter. The absolute dose was measured for the LAO and RPO fields at each position simulated in Pinnacle. The results of the measurements were then compared with Pinnacle calculations.

## III. RESULTS

### A. Planning study

**Figure 3:**
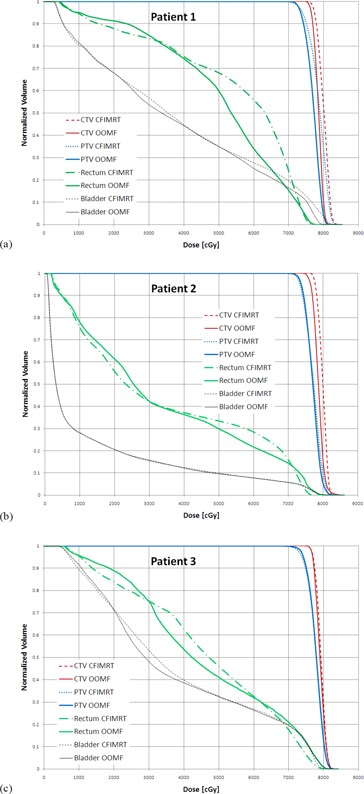
Dose‐volume histograms for CTV, PTV, rectum, and bladder obtained using the CFIMRT and OOMF techniques for patients 1, 2, and 3 are plotted in (a), (b), and (c), respectively.

DVH curves for the CTV, PTV, rectum, and bladder for each patient's CFIMRT and OOMF plan are plotted in Figs. 3(a)–3(c). For clarity, only data from the CFIMRT and OOMF techniques are shown (although in general, data from the OMF and SJOMF techniques tended to bracket the OOMF data, with the OMF and SJOMF techniques resulting in marginally better and poorer OAR sparing, respectively). It is clear that the variation across patients is much greater than the variation across techniques. Note that the dose for all plans was adjusted so that 95% of the prescription dose (or 72.2 Gy) covered at least 98% of the PTV. As a result, the DVH curve corresponding to the CTV is shifted toward higher doses for the CFIMRT plans which cannot achieve the same degree of dose homogeneity as the OOMF plans. Apart from the difference in dose homogeneity to the target, it is the rectal DVH curve that is most affected by choice of technique. For all patients, the volume of rectum receiving between 50 and 60 Gy is reduced with the OOMF technique, whereas the volume of rectum receiving more than 72 Gy is slightly reduced with the CFIMRT technique. Overall, it appears that only patient 1 derives a clinically significant benefit with the OOMF technique (due to the marked reduction in rectal volume receiving more than 60 Gy), while patient 3 may derive a slight benefit with the CFIMRT technique (due to the slight reduction in rectal volume receiving more than 70 Gy). While the OOMF technique appears to reduce the volume of rectum receiving more than 60 Gy for patient 2, that patient's anatomy results in considerable rectal sparing regardless of technique. Finally, the bladder DVH curve appears to be least sensitive to choice of technique.

To understand why patient 3 did not benefit from the OOMF technique, one must consider the dose distribution in detail. In particular, the distance between isodose lines corresponding to 95% and 80% of the prescription dose in the region where the PTV and rectum intersect is about 2 mm shorter with the OOMF technique than with the CFIMRT technique. However, with the CFIMRT technique, the 95% isodose line falls 2 to 4 mm within the posterior border of the PTV, whereas this region of PTV is fully covered by the 95% isodose line with the OOMF technique. Therefore, the volume of rectum within the PTV receives slightly higher dose with the OOMF technique than with the CFIMRT technique, despite the improved dose gradient achieved with the former technique. To complete this discussion, one must also appreciate that the bladder volume of patient 3 (115 cc) is much smaller than that of patient 1 (190 cc) and patient 2 (527 cc). Where the PTV intersects the bladder, the 95% isodose line conforms to the PTV better with the CFIMRT technique than with the OOMF technique. Thus, while both techniques encompass 98% of the PTV with the 95% isodose line, the OOMF technique conforms better to the portion of the PTV adjacent to the rectum, while the CFIMRT technique conforms better to the portion of the PTV adjacent to the bladder for this patient.

**Figure 4:**
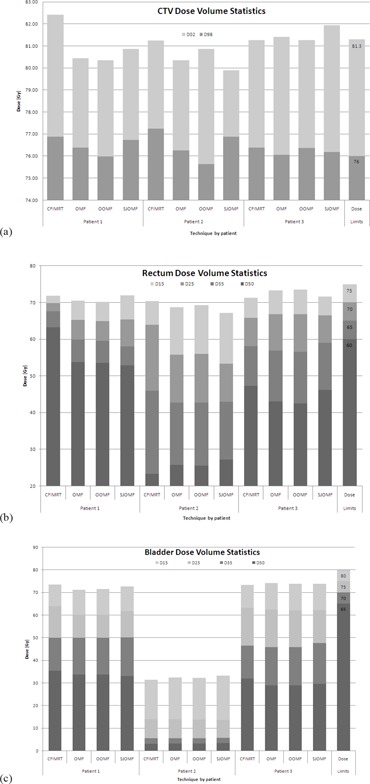
Dose‐volume statistics for each patient and technique investigated are represented for CTV (a), rectum (b), and bladder (c). The right‐hand column indicates the dose objectives used in‐house that are to be met by clinically acceptable plans.

The results for all techniques and patients for selected dose‐volume data points are summarized in Figs. 4(a)–4(c). These particular dose‐volume points are chosen since they comprise the set upon which in‐house plans are judged to be acceptable compared to limits adopted from the RTOG 0126 trial. When considering the dose to the CTV, these criteria were not met for patient 1 using the CFIMRT technique since the maximum dose limit of 107% of the prescription dose (81.32 Gy) to 2% of the CTV volume was exceeded. At the same time, 35% and 50% of the rectal volume of patient 1 exceeded the dose limits of 65 Gy and 60 Gy, respectively, with the CFIMRT technique. Thus it is clear that for patient 1, either an OMF or OOMF technique is favored. With regard to patients 2 and 3, the rectum and bladder dose‐volume criteria were satisfied by all techniques; however, the maximum dose to CTV for patient 3 exceeded the aforementioned limit with the OMF and SJOMF techniques, while the minimum dose to the CTV for patient 2 with the OOMF technique was slightly lower. Therefore, for patients 2 and 3, there is no compelling data to suggest any of the new techniques are favored over CFIMRT.

### B. Dosimetric studies

#### B.1 Dosimetric verification

**Figure 5:**
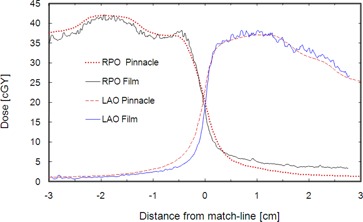
Dose profiles through the isocenter and perpendicular to the match plane corresponding to the LAO and RPO opposed matched field pairs. Results of Pinnacle calculations are compared to GAFCHROMIC film measurements which generally agree with the calculations to within about 5% in the low‐gradient, high‐dose regions, but indicate a steeper gradient at the match line.

Dose profiles obtained from Pinnacle calculation and GAFCHROMIC film measurement through the isocenter and perpendicular to the match plane of the LAO and RPO OMF are plotted in Fig. 5. There is reasonable agreement between Pinnacle calculations and film measurements; however, the measurement shows a sharper penumbra than indicated by calculation. As a consequence, one would expect a higher susceptibility of dose delivery error due to intrafraction motion than that inferred from the Pinnacle model.

#### B.2 Sensitivity to intrafraction motion

**Figure 6:**
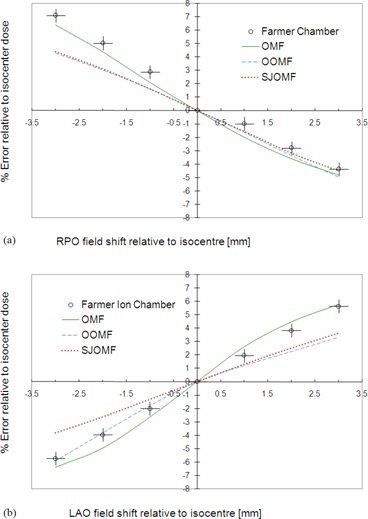
The effect of shifting the isocenter of either the RPO field (a) or the LAO field (b); shifts are perpendicular to the match plane. The dose difference is measured using a Farmer chamber (accurate to within ± 0.5% and positioned to within ±0.2 mm) for the OMF technique only. The discrepancies between calculation and measurement are attributed to partial volume effects in the ion chamber. A reduction in sensitivity to target motion in the direction of the shift is realized when the OOMF or SJOMF techniques are employed.

The Farmer chamber aligned with the isocenter within the phantom was irradiated with a single fraction of an OMF plan using all fields except the RPO field (one field of the OMF pair). The chamber was then shifted in the direction perpendicular to the match plane by various amounts prior to irradiation with the RPO field. The resulting measured and calculated percentage dose difference (from the nonshifted geometry) is plotted in Fig. 6(a) as a function of shift relative to isocenter. The resulting variation in dose represents the maximum error introduced by intrafraction motion per opposed matched field. A corresponding set of measurements and calculations are plotted in Fig. 6(b) where the LAO field (the other field of the OMF pair) was shifted by various amounts in the direction perpendicular to the match plane.

The dose error resulting from this worst‐case shift is determined to be about 2.5% per mm per OMF shifted, whereas a single shift of 2 mm could introduce as much as 5% error to the isocenter dose.

Pinnacle calculation of error due to intrafraction motion across the match plane for the OOMF and SJOMF techniques is also plotted in Figs. 6(a) and 6(b). Among all the junction management techniques, two‐segment feathering employed in the SJOMF technique was least sensitive to small isocenter shifts (about 1.5% per mm shift, per field). By construction, the error is antisymmetric with respect to shift direction since the two forward‐planned segments from each opposed matched field are symmetrically placed about the isocenter. The OOMF technique also reduced the sensitivity, although the error is not antisymmetric with respect to shift direction since the corresponding inverse‐planned field segments of the opposed matched pair are not necessarily distributed symmetrically about the isocenter. Nevertheless, the error reduction is comparable to that realized with the SJOMF technique across much of the range in shifts.

## IV. DISCUSSION

A novel method for treating prostate patients with bilateral metal hip prostheses has been presented and explored. The method employs opposed matched field pairs to allow field incidence from a more lateral direction than is otherwise possible using a conventional IMRT technique. Application of this method to three test patients indicates that it has the potential to reduce dose to rectum and improve dose homogeneity across the PTV. The realization of this potential, however, is found to depend on individual patient anatomy, so it may not necessarily be superior to the conventional technique. Rather, this method presents an alternative approach to planning when the conventional IMRT beam arrangement fails to achieve clinical objectives. While the introduction of opposed matched fields increases the complexity of a plan, the expanded set of adjustable parameters may generally be exploited to yield a plan that meets clinical objectives. Where the conventional plan appears favorable for patient 3, it must be understood that the same set of optimization objectives was used for all techniques. It was not feasible to refine the optimization objectives for each technique individually, although such an approach may address specific compromises presented with each technique.

The laterally‐directed opposed matched fields must each clear the ipsilateral hip prosthesis. This requirement places limits on the amount of prostate displacement in the AP direction that can be tolerated. Gold seed fiducial markers implanted in the prostate are commonly used in conjunction with orthogonal pretreatment imaging to target the prostate. For patients with bilateral MHP, the orthogonal pretreatment images are acquired with the gantry angle displaced by 45° from the vertical and horizontal directions so that the seeds are not occluded by the MHP. Pretreatment imaging and alignment are necessary since interfraction motion of the prostate may be as large as 10–11 mm,^(^ ^19^ ^)^ although shifts greater than 3 mm typically occur in fewer than 5% of the treatment fractions.^(^ ^20^ ^)^ There is the possibility that a laterally‐directed OMF will not clear the MHP once a corrective couch shift is applied. If the magnitude of an AP couch shift correction approaches the margin by which an OMF field clears the MHP, the clearance should be checked using portal imaging. A small adjustment to the gantry angles of the OMF fields may be required to restore clearance. The effect of these adjustments on the dose distribution has not been investigated here, but should be considered for any patient requiring frequent adjustments.

The introduction of match plane raises concern of dose delivery errors for regions near the match plane due to intrafraction motion. Intrafraction prostate motion has been recently investigated.^(^ ^21^ ^)^ The authors found that, over a 4‐minute period, the standard deviation in prostate position for a supine patient was 1.2 mm with a 91.5% probability that the prostate will remain within 2 mm of its initial position. Only the component of motion perpendicular to the match plane contributes to the error associated with field matching. Since the prostate is known to move about equally in the anterior–posterior (AP) and superior–inferior (SI) directions^(^ ^22^ ^)^ and since only the AP motion has a component perpendicular to the match plane, the expected standard deviation in prostate position in the direction perpendicular to the match plane is about 0.6 mm.

To minimize this effect, we recommend that the opposed matched pairs be delivered sequentially in order to minimize the time interval and, therefore, the probability of target motion. In addition, we propose safeguarding against this potential error by adopting an appropriate junction management technique. We investigated SJOMF and OOMF techniques for this purpose. Both techniques significantly reduced the sensitivity of dose delivery to intrafraction motion. SJOMF reduced this error to 1.5% per mm of shift per field, which was the more effective of the two techniques. Furthermore, for patient 2, the SJOMF technique compared favorably with regard to CTV coverage and rectal sparing. Nevertheless, the disadvantage of adopting this technique is two‐fold. First the SJOMF segments must be designed manually, which adds considerable planning time. Second, once the segments needed for shifting the junction are designed, the only parameter that can be optimized for these beams is beam weight. On the other hand, the OOMF technique is simple to implement in Pinnacle since the jaws for the OOMF fields can be set to overlap and be held fixed during the optimization process. Moreover, all the beams undergo IMRT optimization. The resulting feathering in the overlap region is somewhat variable and dependent on how segments are arranged between the two fields. In the plans investigated, segments from both fields contributed approximately equally to the dose in the overlap region. Finally, this technique compares favorably against others with respect to target and organ at risk DVH data for patients 1 and 3, who present a greater challenge to meeting plan objectives than patient 2.

## V. CONCLUSIONS

Based on the three patient plans investigated, we conclude that the OOMF technique is a suitable alternative for clinical use when a satisfactory plan cannot be achieved using a conventional 5‐field IMRT beam arrangement, and that it is preferable to the alternatives we explored in terms of managing intrafraction motion effects on the opposed matched field junction.

## ACKNOWLEDGMENTS

The authors would like to thank the staff at the Juravinski Cancer Centre for providing support during the course of this project. In particular, we would like to thank Randy Passow from the Machine shop for design and construction of the film jig. We also thank the physics assistants, Lisa Gamble, Noel Novo, Thuy Lam, Laura Hagey, and Daniel Sopher, for their guidance during the measurements.
